# An Object Hiding Behind the Screen: The Relationship Between Body Surveillance and Internet Addiction Among Chinese Women

**DOI:** 10.1007/s10508-026-03442-0

**Published:** 2026-05-25

**Authors:** Yufei Jiang, Natalie Hiu-Lam Wong, Hill-Son Lai, Ying Chuen Chan, Kai-Tak Poon

**Affiliations:** 1 Department of Administrative Management, School of Law, Hangzhou City University, Hangzhou, 310015 China; 2 Department of Psychology and Centre for Psychosocial Health, The Education University of Hong Kong, Hong Kong, China

**Keywords:** Body surveillance, Self-objectification, Appearance anxiety, Self-control, Internet addiction

## Abstract

Women often monitor their bodies to meet societal standards of beauty. However, researchers have yet to test how this tendency may be associated with various psychological problems, especially those related to computer and Internet usage. In the current study, we examined whether body surveillance may predict Internet addiction and whether appearance anxiety and self-control may account for the proposed link. A total of 448 Chinese female undergraduates in Hong Kong completed validated measures assessing body surveillance, appearance anxiety, self-control, and Internet addiction. Regression analyses revealed that body surveillance was positively associated with appearance anxiety and Internet addiction and was negatively associated with self-control. Moreover, structural equation modeling and Monte Carlo simulation showed that the relationship between body surveillance and Internet addiction was serially mediated by appearance anxiety and self-control. These findings advance current theories and knowledge by demonstrating that body surveillance may predict Internet addiction and unveil the underlying psychological mechanisms. Finally, we discuss implications for theories of body surveillance and Internet addiction and implications for educators and practitioners in the hopes of enhancing women’s health regarding body surveillance and Internet addiction, as well as their well-being.

## Introduction

In urban areas of modernized societies, we are inundated with physical and digital forms of advertisement. Particularly for the clothing and beauty industries, pictures illustrating scantily clad female models might draw passersby’s attention. To chase after these “standards” of beauty, women may engage in active surveillance of their bodies to resemble the media portrayals (Aubrey et al., 2009; Butkowski et al., 2019). Studies have enriched our understanding of the adverse impacts of *body surveillance*, which refers to one’s habitual self-consciousness of their outward appearance and body (McKinley & Hyde, 1996). For example, research has shown that body surveillance predicts loneliness (Teng et al., 2019), social anxiety (Teng & Poon, 2020), body shame (Sun, 2018; see Saunders et al., 2024 for a review), and depressive symptoms (Chan et al., 2024; Milan & Perez, 2021).

Although researchers have shown various psychological and behavioral outcomes of body surveillance, few have tested whether it predicts people’s Internet usage. The existing literature has begun contributing to our understanding of the relationship between body surveillance and social media usage (e.g., Couture Bue, 2020; Manago et al., 2015; Yu et al., 2023) and the impacts of taking and posting selfies (e.g., Choukas-Bradley et al., 2019; Salomon & Brown, 2021); however, few researchers have examined whether body surveillance may predict Internet addiction despite the severe problems it may pose to women’s mental health.

The Internet is an integral part of modern civilization because people use it in broad aspects of life for multiple purposes (Kappeler, 2024; Yellowlees & Marks, 2007), such as logistical communication (e.g., completing work-related tasks in a company), entertainment (e.g., gaming and watching movies), and socializing (e.g., maintaining relationships and making new friends). The fast-growing nature of the Internet can enhance our quality of life and offer many benefits. However, overreliance on these technologies could be problematic. Internet addiction is defined as the lack of ability to regulate the amount of time and energy spent on the Internet, resulting in various social and cognitive consequences that affect daily functioning (Davis, 2001; Young, 1996). It is associated with wide-ranging detriments to one’s psychological and physical well-being as well as interpersonal, occupational, and social functioning (e.g., Chau et al., 2024; Duke & Montag, 2017; Kuss & Lopez-Fernandez, 2016; Saadati et al., 2021; Seki et al., 2019). With Internet addiction being a global health problem (Dong et al., 2020; Lozano-Blasco et al., 2022; Zuckerman, 2020), it is crucial to test the potential contributing factors of Internet addiction and identify their corresponding psychological mechanisms. Therefore, in the current study, we examined whether body surveillance predicts Internet addiction and investigated whether appearance anxiety and self-control serially mediated the relationship between body surveillance and Internet addiction.

### Body Surveillance and Internet Addiction

Body surveillance is associated with experiences of sexual objectification (Lin et al., 2023; Szymanski, 2020), defined as the reduction of an individual to a collection of body parts, appearance, or sexual function (Chan & Poon, 2025; Szymanski et al., 2011). Women encounter these objectifying experiences, which are associated with behavioral (Poon & Jiang, 2020) and mental health problems (Jiang et al., 2022), approximately every other day (Holland et al., 2017), suggesting that the experience of body surveillance may be a particularly pressing issue for women. According to objectification theory (Fredrickson & Roberts, 1997), objectifying events lead women to internalize others’ views and evaluations of their bodies, thereby viewing themselves as objects from others’ perspectives in a process termed *self-objectification*. By adopting this perspective, objectified women internalize the cultural body standards, requiring them to monitor their bodies chronically to ensure they comply with these standards (McKinley & Hyde, 1996). Therefore, self-objectification has been found to be associated with body surveillance (e.g., Jongenelis & Pettigrew, 2020; Vandenbosch & Eggermont, 2012).

Researchers have also treated body surveillance as a manifestation of self-objectification and used the measure of body surveillance to reflect self-objectification (Daniels et al., 2020; Jones & Griffiths, 2015). Theories on the relationship between body surveillance and Internet usage have been mixed. Some scholars have proposed that excessive use of social media may promote body surveillance because the increased exposure to appearance-related conversations and pictures on social media platforms incites the comparison to ideal or others’ appearance, thereby increasing body image concerns (see Fardouly & Vartanian, 2016 for a review). In contrast, other scholars have theorized that body image concerns may precede problematic media use because people who are dissatisfied with their appearance can strategically manage their self-presentation by editing and posting their selfies on social media, which may predict problematic social media use (Boursier et al., 2020). Given that social media use represents only one use of the Internet (Tereshchenko, 2023; Yellowlees & Marks, 2007), problematic social media use may be connected to Internet addiction but not necessarily equivalent to it. Although the research mentioned above has revealed a bidirectional relationship between body surveillance and social media addiction, body surveillance may be related to other uses of the Internet, as well. However, the relationship between body surveillance and Internet addiction remains understudied.

Women with high body surveillance actively regulate their attention and direct it toward their appearance. The continuous exertion of self-regulation leads people to fatigue (Lin et al., 2020). Body surveillance requires significant effort when one monitors their appearance and considers how their body looks to others, which, theoretically, comes at the cost of depleted cognitive resources (Quinn et al., 2011). Because self-regulation depends on these cognitive resources (Inzlicht & Schmeichel, 2012; Kim & Maglio, 2025), the decrease in cognitive resources following body surveillance may ultimately result in less self-regulatory behaviors. Research has revealed that body concerns are associated with behaviors characterized as failures of self-regulation, such as binge eating (Mehak et al., 2018), drinking motives (Baildon et al., 2021), cigarette smoking (Fiissel & Lafreniere, 2006; Napolitano et al., 2020), and self-harm (Ren et al., 2024; Nelson & Muehlenkamp, 2012).

Internet addiction shows the characteristics of compulsive–impulsive spectrum disorder and is considered an impulse disorder (Zhou et al., 2010). Therefore, body surveillance, which depletes cognitive resources and worsens self-regulatory performance, may be linked to Internet addiction. Empirical research has revealed a significant association between body concerns and problematic social media use (e.g., Boursier et al., 2020; Gioia et al., 2020), which provides evidence supporting the relationship between body surveillance and Internet addiction. Indeed, research has shown that a high portion of women feel concern for their bodies and perceive their bodies as unattractive (Luo et al., 2005). Considering the high prevalence of body concerns among women and their close associations with worse self-regulatory behaviors, in the present study, we aimed to show the relationship between body surveillance and Internet addiction and the underlying psychological mechanisms. Specifically, we predicted that the relationship between body surveillance and Internet addiction was serially mediated by appearance anxiety and self-control.

### The Relationship Between Body Surveillance and Appearance Anxiety

As stated in the objectification–social comparison model (Seekis et al., 2020), body surveillance proceeds and manifests as appearance anxiety. Appearance anxiety is a specific fear of negative evaluations based on aspects of one’s appearance, such as physique (e.g., height, weight, and muscle tone) and facial features, resulting in constant insecurity about outward display (Dion et al., 1990; Hart et al., 1989). The objectification–social comparison model proposes that body surveillance is based on upward social comparison. When women with high body surveillance chronically think about and monitor their bodies, they are comparing their appearance with the cultural ideal appearance. The ideal appearance is often unattainable because beauty standards vary across cultures and socioeconomic status (Swami et al., 2010), and individuals may readily distort their perceptions of their own body images (Heinberg, 2001).

In addition, the tendency for women to engage in body surveillance is often irrelevant to their actual body image or objective attractiveness. For example, body mass index is not significantly associated with levels of body surveillance (Wollast et al., 2020). Consequently, women with high levels of body surveillance are susceptible to the upward comparative process, regardless of variations in attractiveness or appearance. Comparing oneself with a superior target tends to threaten people’s self-esteem and lead to worse affective outcomes (Gerber et al., 2018), including increased feelings of anxiety (e.g., Butzer & Kuiper, 2006; Jiang & Ngien, 2020). The upward social comparison of appearance is related to a sense of inferiority (Szymanski, 2020). It makes women feel less satisfied with their appearance and worry more about how others assess it (Strahan et al., 2006). Therefore, frequent surveillance of one’s body may lead to chronic upward comparison with ideal appearance, resulting in worse appearance self-regard and more appearance anxiety.

Indeed, research has provided evidence showing the link between body surveillance and appearance anxiety. For example, body surveillance has been linked to female undergraduates’ appearance anxiety in their daily lives (Steer & Tiggemann, 2008) and appearance-based rejection sensitivity (Teng & Poon, 2020). Similarly, findings have also shown that body surveillance is associated with appearance anxiety in sexual situations (Vencill et al., 2015). In addition, individuals who heavily emphasize appearance and actively engage in grooming behaviors are more vulnerable to appearance anxiety (Hart et al., 2008). Research has also shown that when young women are induced to surveil their appearance by taking and posting selfies, they experience higher levels of anxiety and are preoccupied with concerns over their physical appearance (Mills et al., 2018). Based on these theoretical and empirical accounts, we predicted that body surveillance was positively associated with appearance anxiety.

### Appearance Anxiety Explicates the Relationship Between Body Surveillance and Self-Control

According to the pleasure principle (Laplanche & Pontalis, 2018), people often desire immediate pleasures and benefits that may violate personal and societal norms or standards. However, humans are obligated to fulfill social order to avoid punishment (Gross & Vostroknutov, 2022). Therefore, people need to possess self-control, which refers to intentionally altering one’s desired responses to meet social standards (Baumeister et al., 1994; Baumeister & Vohs, 2016). The activation of self-control requires people to spend additional effort to override the impulses and desires that promote immediate gratification (Gillebaart, 2018). For example, athletes need to follow strict diets and exercise routines by resisting unhealthy food and rest; students may need to override their desires to hang out with friends during exam periods to reserve time for studying. As mentioned above, studies have shown that body surveillance is associated with adverse consequences of lacking self-control, including binge eating, alcohol consumption, cigarette smoking, and self-harm (Baildon et al., 2021; Fiissel & Lafreniere, 2006; Mehak et al., 2018; Nelson & Muehlenkamp, 2012; Oliver et al., 2024; Schaefer et al., 2018; Zheng & Lyu, 2025). In the present study, we proposed that appearance anxiety might explicate the link between body surveillance and self-control.

A review of self-control models suggests that emotion often interferes with self-control and demotivates people’s efforts to retain self-control (Inzlicht et al., 2021). One of the reviewed models, the strength model (Muraven & Baumeister, 2000), asserts that resources for self-control are limited and exerting limited resources on specific tasks may impair subsequent attempts at self-control. Women with high appearance anxiety may spend extra effort in striving for a better appearance, thereby compromising their self-control resources. For example, appearance anxiety is associated with women’s interest in cosmetic surgery (Gillen & Markey, 2021). Moreover, women who experience body image disturbances often participate in maladaptive eating behaviors to control their body shapes, such as decreasing water consumption and employing appetite-suppressing strategies (Jongenelis & Pettigrew, 2020; Langdon & Dennee-Sommers, 2010). After allocating excessive resources to appearance-related domains, these appearance-anxious women may find themselves lacking resources to attend to other areas, such as their health, personal growth, and development of interpersonal skills (Swim et al., 1995). As a result, they lack sufficient self-regulatory resources to control their desires voluntarily when facing temptations, thereby suffering from impaired self-control. Thus, appearance anxiety may mediate the link between body surveillance and self-control.

Although researchers have not directly tested the proposed mediation relationship, indirect evidence has suggested the potential associations between body surveillance, appearance anxiety, and impaired self-control. In one experimental study, women who were experimentally induced to monitor their appearance (e.g., by wearing a swimsuit) reported higher levels of appearance anxiety and subsequently performed worse on physical tasks that required focus and self-control (Dimas et al., 2021); however, the mediating role of appearance anxiety in the relationships between body surveillance and task performance remains unexamined. Another study has shown that appearance anxiety is negatively associated with self-control (Çelik & Turan, 2014). Moreover, appearance anxiety is positively associated with various forms of addiction (e.g., video gaming addiction; Dindar & Akbulut, 2015) and excessive behavior (e.g., binge eating; Brosof & Levinson, 2017), which are related to a lack of self-control (Pearson et al., 2018; Walters et al., 2018; also see review in De Ridder et al., 2012). In sum, based on the theoretical and empirical evidence, we predicted that appearance anxiety mediated the relationship between body surveillance and impaired self-control.

### Implications for Internet Addiction

In the present study, we further tested whether impaired self-control carried direct implications for Internet addiction. Regular practices of small acts inhibiting moods, urges, thoughts, or feelings could increase self-control (Muraven & Baumeister, 2000). High levels of self-control are often associated with various desirable outcomes, such as better interpersonal relationships (Liu & Li, 2020), greater decision-making satisfaction (Kokkoris et al., 2019), and effective refraining from addictive behaviors (Baumeister & Vonasch, 2015). In contrast, impaired self-control is associated with the experience of many adverse consequences, such as procrastination (Kim et al., 2017), dishonesty (Poon et al., 2023), being prone to alcohol and drug addiction (e.g., Remmerswaal et al., 2019; Sripada, 2022; Walters et al., 2018), and increased aggression (Osgood & Muraven, 2016). Self-control can also enhance one’s cognitive functioning and the development of healthy lifestyle habits, including advancement in academic and work performance (Duckworth et al., 2019) and physical activity participation (Hagger et al., 2019).

In this cyber age, in which technology and social media are highly accessible, self-control in Internet use has raised great research interest in recent years. Stemming from the well-known cognitive–behavioral model of generalized problematic Internet use (Caplan, 2010), the deficit in self-control is a direct predictor of Internet addiction because it leads to failure in monitoring, judging, and adjusting Internet use. In addition, the model proposes that psychological problems, such as anxiety, may predispose individuals to seek the Internet to alter their negative affective states and, in turn, regulate their mood because they can seek connections with others through the Internet and engage in Internet activities that make them feel better (Caplan, 2002). However, this behavior usually results in adverse outcomes because diminished self-control leads to maladaptive Internet-related cognitions and behaviors, such as obsessive thoughts about using the Internet and difficulties in reducing time spent on the Internet. Whereas self-control enables people to focus on events leading to long-term benefits rather than attending to immediate gratification (Inzlicht et al., 2021), those lacking self-control may use the Internet to relieve their appearance anxiety and gain instant mood enhancement. Despite these temporary benefits, this use of the Internet may worsen individuals’ interpersonal relationships and offline social participation because Internet addiction has considerable costs in terms of time spent on face-to-face social engagements and other in-person activities.

**Fig. 1:**
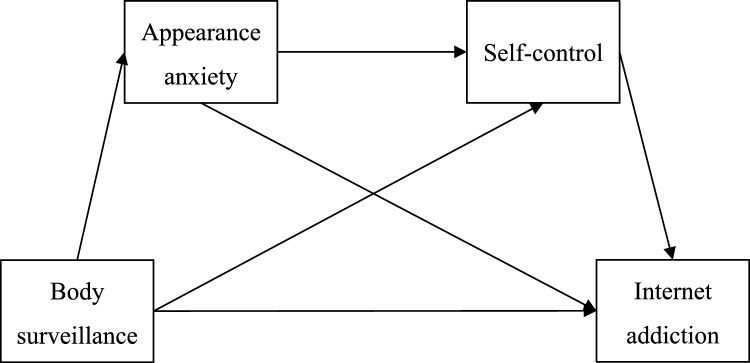
Conceptual model of the current study

Research has provided abundant empirical evidence showing that impaired self-control is a critical predictor of Internet addiction. For example, cross-sectional studies have shown that self-control is negatively correlated with Internet addiction (e.g., Li et al., 2014; Poon, 2018). Moreover, longitudinal studies have further established the temporal sequence by showing that impaired self-control can predict subsequent Internet addiction (e.g., J. Li et al., 2021a, 2021b; Yu & Shek, 2018). A recent meta-analysis of 83 studies also revealed a robust relationship between self-control and Internet addiction (S. Li et al., 2021a, 2021b). Therefore, the existing literature has established a solid basis, suggesting that self-control should be negatively associated with Internet addiction. We proposed in our theoretical model that women with higher body surveillance should have higher levels of appearance anxiety and lower levels of self-control and, in turn, be at greater risk of Internet addiction. Therefore, we predicted that the relationship between body surveillance and Internet addiction was serially mediated by appearance anxiety and self-control (Fig. 1).

## Method

### Participants and Procedure

We recruited participants from a public university in Hong Kong, China, through a mass email. They would receive a small monetary reward for their participation. Eligibility criteria required participants to identify themselves as female, undergraduate, and Chinese. Participants completed a computerized survey in person at a laboratory on campus. To prevent missing responses, the survey system would prompt participants to answer any unanswered questions, thus ensuring no missing data at the item level. Before participating in the study, participants were verbally informed that they could withdraw from the study at any time without facing any negative consequences. This point was also clearly written in the consent form. They would still receive the compensation if they decided to withdraw from participating in the study. A research assistant was available to provide immediate support for any withdrawal requests and provide compensation.

A total of 458 women completed the study. Of those participants, 10 participants failed to pass the attention check question and were excluded from the data analyses (e.g., Oppenheimer et al., 2009; Poon & Wong, 2021). Including these participants in the analyses would not significantly affect the results because the coefficients and model fits obtained were almost identical to those reported below. The final sample that passed the attention check consisted of 448 women between 18 and 29 (*M*_age_ = 20.63, *SD*_age_ = 2.10). Regarding religious beliefs, the majority were nonreligious (75.4%), 16.3% were Christians, 2.5% were Buddhist, 0.9% were Catholic, and the rest were other religions (4.9%).

### Measures

#### Body Surveillance

Participants completed the Surveillance subscale from the Objectified Body Consciousness Scale (McKinley & Hyde, 1996). They indicated their agreement with eight items, such as, “During the day, I think about how I look many times” and “I often worry about whether the clothes I am wearing make me look good,” on a 7-point scale (1 = *strongly disagree*, 7 = *strongly agree*). Scores were reverse-coded when necessary and summed to indicate body surveillance, with higher scores indicating higher levels of body surveillance (Cronbach’s *α* = .75).

#### Appearance Anxiety

Participants completed the brief version of the Appearance Anxiety Scale (Dion et al., 1990). They rated how frequently they experienced or enacted the thoughts and behaviors in each of the 14 items on a 5-point scale (1 = *never*, 5 = *almost always*). Sample items include “I feel nervous about aspects of my physical appearance” and “I feel uncomfortable with certain aspects of my physical appearance.” Scores were summed to indicate appearance anxiety, with higher scores representing greater appearance anxiety (Cronbach’s *α* = .86).

#### Self-Control

Participants completed the brief version of the Self-Control Scale (Tangney et al., 2004). They rated the degree to which each of the 13 items reflected how they typically were on a 5-point scale (1 = *not at all*, 5 = *very much*). Sample items include “I am good at resisting temptation” and “I refuse things that are bad for me.” Scores were reverse-coded when necessary and summed to indicate self-control, with higher scores representing higher levels of self-control (Cronbach’s *α* = .85).

#### Internet Addiction

Participants completed the Internet Addiction Test (Young, 1998). This test has been translated and adapted into multiple languages and shown good reliability and validity in various populations (e.g., Boysan et al., 2017; Lai et al., 2013; Tsimtsiou et al., 2014), supporting its use in the present study with a Chinese sample. It is one of the most commonly used scales for Internet addiction in research (Sánchez-Fernández et al., 2022), facilitating the comparison of the present results with existing findings. The scale comprises six factors of Internet addiction: salience, excessive use, neglecting work, anticipation, lack of control, and neglecting social life (Widyanto & McMurran, 2004). Participants indicated their Internet use patterns and risks of Internet addiction by responding to 20 items, such as, “Do you find that you stay on-line longer than you intended?” and “Do you choose to spend more time on-line over going out with others?” on a 5-point scale (1 = *does not apply or rarely*, 5 = *always*). Scores were summed to indicate participants’ Internet addiction, with higher scores representing more severe Internet addiction (Cronbach’s *α* = .91).

### Data Analytic Strategy

We tested the hypothesized serial mediation model using structural equation modeling (SEM) in the lavaan package (Rosseel, 2012) for the R Environment (R Core Team, 2017). As in previous studies (e.g., Widyanto & McMurran, 2004), we treated Internet addiction as a latent variable, with salience, excessive use, neglecting work, anticipation, lack of control, and neglecting social life as six factors of it. The goodness of fit was indicated by the ratio of $\upchi$ ^2^/*df* below 5 (Schumacker & Lomax, 2004), the values of the comparative fit index (CFI) and Tucker–Lewis index (TLI) above .95, the root-mean-square error of approximation (RMSEA) value below .08, and the standardized root-mean-square residual (SRMR) value below .06 (Hu & Bentler, 1999). In addition, we used the semTools package (Jorgensen et al., 2018) to conduct the Monte Carlo simulation with 20,000 resamples to construct the confidence intervals (CIs) for the mediating effects (Preacher & Selig, 2012). A Monte Carlo-simulated CI excluding zero indicates a significant mediating effect.

## Results

### Relationships Between Body Surveillance, Appearance Anxiety, Self-Control, and Internet Addiction

Table 1 shows the descriptive statistics and bivariate correlations for the variables of interest. We first conducted a series of simple regression analyses. The results revealed that body surveillance was positively associated with appearance anxiety, *B* = .62, $\beta$ = .47, *SE* = .05, *p* < .001, and negatively associated with self-control, *B* = −.30, $\beta$ = −.25, *SE* = .06, *p* < .001. Moreover, body surveillance was positively associated with Internet addiction, *B* = .58, $\beta$ = .27, *SE* = .10, *p* < .001.

**Table 1:** Descriptive statistics and correlations for study variables

Variable	1	2	3	4
1. Body surveillance	–			
2. Appearance anxiety	.47^***^	–		
3. Self-control	−.25^***^	−.43^***^	–	
4. Internet addiction	.27^**^	.41^***^	−.47^***^	–
*M*	36.18	44.28	36.45	47.85
SD	6.64	8.64	8.01	14.06
Range	12–56	22–69	14–57	20–91
Scale range	8–56	14–70	13–65	20–100

^***^p < .001

### Structural Equation Modeling and Mediation Analysis

**Fig. 2:**
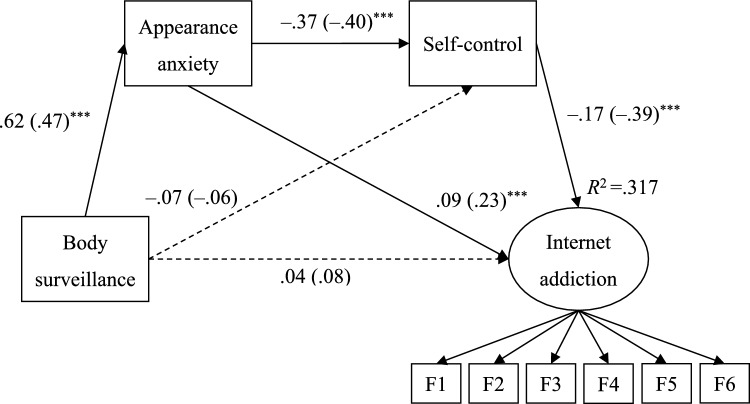
Serial mediation model of the association between body surveillance and Internet addiction. *Note.* The association between body surveillance and Internet addiction was serially mediated by appearance anxiety and self-control. Statistics outside parentheses are unstandardized coefficients; statistics inside parentheses are standardized coefficients. Dotted lines represent nonsignificant direct paths. F1 to F6 refer to the six factors measured in the Internet addiction scale: salience, excessive use, neglecting work, anticipation, lack of control, and neglecting social life (Widyanto & McMurran, 2004). ^***^p < .001

Our model examined whether the relationship between body surveillance and Internet addiction is serially mediated by appearance anxiety and self-control (Fig. 2). The six dimensions of Internet addiction were significantly loaded on the latent Internet addiction variable (ps < .001). The results supported our predicted serial mediation model because all the indices indicated good model fit, *χ*^2^/*df* = 3.55, CFI = .967, TLI = .950, RMSEA = .075, SRMR = .032. Moreover, the model accounted for 31.7% of the variance in Internet addiction (*R*^2^ = .317). The total association between body surveillance and Internet addiction without controlling for the two mediators, appearance anxiety and self-control, was significant, *B* = .15, $\beta$ = .28, *SE* = .03, *p* < .001. After controlling for the other two variables through direct analyses, Internet addiction was not significantly predicted by body surveillance, *B* = .04, $\beta$ = .08, *SE* = .03, *p* = .101, but it was significantly predicted by appearance anxiety,* B* = .09, $\beta$ = .23, *SE* = .02, *p* < .001, and self-control,* B* = −.17, $\beta$ = −.39, *SE* = .02, *p* < .001.

Furthermore, the results of the Monte Carlo simulation with 20,000 resamples showed significant mediating effects. The association between body surveillance and self-control was significantly mediated by appearance anxiety, *B* = −.23, $\beta$ = −.19, *SE* = .03, *p* < .001, 95% CI [−.30, −.17], indicating appearance anxiety mediated the relationship between body surveillance and self-control. In addition, the association between body surveillance and Internet addiction through appearance anxiety and self-control was significant, *B* = .04, $\beta$ = .07, *SE* = .01, *p* < .001, 95% CI [.03, .06]. This finding indicated that appearance anxiety and self-control serially mediated the association between body surveillance and Internet addiction.

### Alternative Models

Previous studies found that appearance anxiety may precede body surveillance (Keelan et al., 1992), and body surveillance may precede Internet addiction (see the review in Fardouly & Vartanian, 2016). Therefore, there may be alternative pathways that can explain the link between body surveillance and Internet addiction. To account for this, we tested two alternative serial mediation models.

We first tested the appearance anxiety–body surveillance–self-control–Internet addiction model. However, the results indicated that the model did not fit well, *χ*^2^/*df* = 508.17, CFI = .098, TLI = −.240, RMSEA = 1.064, SRMR = .111, and the serial mediation was not significant, *B* = .004, $\beta$ = .01, *SE* = .004, *p* = .224, 95% CI [−.003, .01]. We also tested the self-control–Internet addiction–body surveillance–appearance anxiety model. The results showed that the model fit well, *χ*^2^/*df* = 3.55, CFI = .967, TLI = .950, RMSEA = .075, SRMR = .032. The model accounted for 35.9% of the variance in appearance anxiety (*R*^2^ = .359). In addition, the serial mediation was significant, *B* = −.04,$\beta$ = −.04, *SE* = .01, *p* = .001, 95% CI [−.07, −.02]. Aligned with previous findings (Wang et al., 2021), the present findings suggested a bidirectional relationship between body surveillance and Internet use. We discussed the alternative models in the Limitations and Future Research Directions section.

## Discussion

With the advancements in technology, every person is only a touch away from experiencing the omnipresence and information explosion of the Internet (Mackey & Jacobson, 2011). The proper use of the Internet can bring convenience and enhance human communication. However, when Internet use is abused and uncontrolled, significant problems for individual and societal well-being may arise (Kamolthip et al., 2022; Kato et al., 2020). Therefore, it is important to examine factors that may predict Internet addiction. Our theoretical model posited that body surveillance, which is a practice found predominantly in young women, may predict Internet addiction, whereas appearance anxiety and self-control may serve as the psychological mechanisms underlying the link. The current results provided support to our predictions. Women with higher body surveillance reported higher levels of appearance anxiety, lower levels of self-control, and higher tendencies toward Internet addiction. Moreover, the relationship between body surveillance and Internet addiction was serially mediated by appearance anxiety and self-control.

The current research has revealed a significant relationship between body surveillance and appearance anxiety. It adds support to the existing theories of objectification. For example, the objectification theory (Fredrickson & Roberts, 1997) and the integrated objectification–social comparison model (Seekis et al., 2020; Tylka & Sabik, 2010) posit that body surveillance proceeds appearance anxiety. In the current study, we used structural equation modeling to examine the body surveillance–appearance anxiety path and the reversed path. The results showed that the former model fit well and the latter did not. However, the sequential inference for the body surveillance–appearance anxiety path requires further experimental or longitudinal studies.

The present findings dovetail nicely with previous work demonstrating the relationship between body surveillance and the tendencies to engage in impulsive and addictive behaviors. For example, in those studies, women with high body surveillance tended to participate in binge eating behaviors (Mehak et al., 2018) and exhibit aggressive and antisocial tendencies (Agbaria, 2021). Extending these findings, the current study reveals the association of body surveillance with another maladaptive behavior, Internet addiction. Taken together, the present study contributes to our understanding of the wide range of maladaptive behaviors that may be related to body surveillance, suggesting the pernicious nature of sexual objectification.

Psychosocial problems are theorized to be linked to Internet addiction (see Caplan, 2002, 2003, 2010; Davis, 2001). By showing body surveillance and appearance anxiety as potential risk factors for Internet addiction, the present study provides additional empirical evidence supporting the relationship between psychosocial problems and Internet addiction. The Internet has become a conventional approach for modern people to cope with negative experiences (McNicol & Thorsteinsson, 2017). Specifically, in the present study, we found that women who surveilled their bodies more frequently were more likely to be addicted to the Internet. The present findings echo previous empirical research that shows Internet addiction is more predominant among people with psychosocial problems, such as loneliness (Özdemir et al., 2014), deficits in recognizing facial expressions (Chen et al., 2017), ostracism (Poon, 2018), and social anxiety (Baltaci et al., 2021).

The present study also advances current knowledge by identifying the psychological mechanisms that explain the relationship between body surveillance and Internet addiction. In particular, the current study showed the crucial role of affect (i.e., appearance anxiety) and self-control in elucidating the relationship between body surveillance and Internet addiction. Whereas previous research has mainly hypothesized the relationship between body concerns and social media addiction based on the self-presentational theory (i.e., online contexts enable people to manage and control personal images; Boursier et al., 2020; Gioia et al., 2020), the present study links body surveillance and appearance anxiety to a broader form of addiction (i.e., Internet addiction) through the lens of self-control models (Inzlicht et al., 2021).

### Implications of the Current Research

The potential negative psychological and behavioral outcomes associated with body surveillance found in the current study underscore the need to reduce excessive focus on bodies and physical appearance. In this study, female college students with high body surveillance tendencies are more likely to experience appearance anxiety, face impaired self-control, and exhibit a higher risk of Internet addiction. Importantly, both adult women and adolescent girls often develop body surveillance behaviors, as indicated in previous studies (e.g., McKenney & Bigler, 2016; Vandenbosch & Eggermont, 2012). These patterns reflect broader systemic issues influenced by cultural norms and media portrayals, rather than merely personal traits and attributes. For example, adolescent girls and adult women often encounter images of women in minimal clothing and poses that accentuate their bodies on Instagram. This persistent exposure may lead them to internalize thin ideals, which subsequently increases their tendencies toward body surveillance (Skowronski et al., 2022).Therefore, addressing body surveillance requires both individual support and systemic change.

At the individual level, educators and parents may help young students avoid excessive body surveillance by promoting body positivity and acceptance, increasing media literacy regarding body ideals, and encouraging healthy self-care practices. Previous research has shown that sexually objectifying behaviors and media representations in real and virtual contexts contribute to increased body surveillance and body image concerns (Fardouly & Vartanian, 2016; Kleemans et al., 2018; Krawczyk & Thompson, 2015; Szymanski, 2020), Therefore, on a systemic level, a fundamental change in body surveillance requires addressing underlying sociocultural issues, such as challenging unrealistic media representations and advocating for the value and acceptance of bodies of all sizes, which aligns with the health-at-every size paradigm (Bombak, 2014).

The observed mediating roles of appearance anxiety and self-control may provide healthcare professionals with practical insights for developing intervention strategies to mitigate the negative consequences of body surveillance and Internet addiction. Specifically, to weaken the link between body surveillance and Internet addiction, practitioners may focus on reducing patients’ appearance anxiety or enhancing their self-control. To help decrease appearance anxiety, practitioners can provide patients with training options, such as compassion-focused self-help (Hudson et al., 2020), psychosocial interventions (Clarke et al., 2013), and acceptance and commitment therapy (Shepherd et al., 2020). To enhance self-control, they can implement interventions, such as cognitive-behavioral therapy (Agbaria, 2022) and mindfulness practices (Liu et al., 2022). This proposal aligns with previous research indicating that lowering appearance anxiety (Baltaci et al., 2021) and boosting self-control (Agbaria, 2022) can be effective strategies for reducing Internet addiction. Nevertheless, the effectiveness of using these intervention strategies in addressing women’s Internet addiction associated with appearance-monitoring behaviors still requires further empirical evidence.

### Limitations and Future Research Directions

Several limitations within this study are proposed for future research to consider. First, due to the study’s cross-sectional nature, we are unable to draw causal conclusions with the present findings. One previous study found that appearance anxiety in late adolescence and early adulthood may stem from negative social experiences in childhood and early adolescence, such as negative comments about appearance (Keelan et al., 1992). Hence, appearance anxiety may precede body surveillance. In addition, researchers have proposed that social media addiction leads to body surveillance (see the review in Fardouly & Vartanian, 2016). Therefore, there may be alternative pathways that account for the link between body surveillance and Internet addiction. To account for this, we tested two alternative serial mediation models (i.e., the appearance anxiety–body surveillance–self-control–Internet addiction path and the self-control–Internet addiction–body surveillance–appearance anxiety path). The results showed that the former serial mediation was not significant, but the latter was significant. Although our theoretical model was derived from prevailing theories and empirical findings (e.g., Caplan, 2010; Davis, 2001), future studies could establish the causal relationships between the studied variables using experiments and investigate their long-term impacts using longitudinal methodologies.

Second, our study did not measure the different manifestations of Internet addiction. People spend their time online on various activities, such as social networking, reading the news, and researching. As stated previously, Internet addiction is a broad and general form of problematic Internet use (Yellowlees & Marks, 2007). It has distinct manifestations, such as excessive gaming, cybersexual addiction, and problematic social media use (Block, 2008). Existing research has provided evidence supporting that body surveillance links to problematic social media use (Boursier et al., 2020; Gioia et al., 2020). Researchers may continue expanding on our theoretical model by examining whether body surveillance predicts maladaptive Internet use in other specific domains.

Third, in addition to appearance anxiety and self-control, there might be other psychological processes that influence the relationship between body surveillance and Internet addiction deserving further investigation. For example, research has shown the desire to control one’s body image as the mediator in the relationship between body shame and social media use (Gioia et al., 2020). Therefore, it may also mediate the relationship between body surveillance and Internet addiction. In addition, reduced self-esteem and self-efficacy, increased negative affect, and an avoidant coping strategy after body surveillance may be linked to Internet addiction (Baltaci et al., 2021; Jones & Griffiths, 2015; Veldhuis et al., 2020). Researchers may test whether these psychological factors mediate the relationship between body surveillance and Internet addiction. Relatedly, future research may also examine whether certain dispositional factors may moderate this relationship. For instance, high trait self-compassion (Liss & Erchull, 2015; Wollast et al., 2020), grandiose narcissism (Carrotte & Anderson, 2019), and feminist identity (Murnen & Smolak, 2009) have been proposed to protect women from the adverse outcomes of body concerns. By identifying additional psychological mechanisms and revealing moderating factors, we can further expand our scope of knowledge, advance existing theories, and facilitate the design of intervention strategies.

Fourth, we focused on a female sample in the present study. Compared to women, men are less likely to surveil and ruminate about their bodies (Wang et al., 2020), and their body surveillance tendency is associated with fewer psychological symptoms (Hyde et al., 2008). Regarding Internet addiction, there are mixed results on the prevalence of Internet addiction among men and women, and men tend to engage in dominant activities online, while women prefer activities that hide their appearance online (Chou et al., 2005; Kuss et al., 2014). Given these gender differences in the responses to body surveillance and patterns of Internet addiction, further research is needed to test whether the present findings also apply to men.

Finally, in our study, we utilized a survey system that would prompt participants to answer any unanswered questions, aiming to minimize item nonresponse rates. This approach has been recommended by several researchers and has demonstrated a significant reduction in item nonresponse rates without impacting dropout rates or participants’ motivations to participate (e.g., Albaum et al., 2010, 2011; 2014; De Leeuw et al., 2015; Roster et al., 2014). However, this approach may increase the likelihood of dishonest or socially desirable responses (Dillman et al., 2014; Stieger et al., 2007). To mitigate these potential concerns, future studies may consider including “prefer not to answer” options or allowing participants to leave some items blank.

### Conclusion

The present study addresses a gap in the literature by investigating the relationship between body surveillance, a common manifestation of self-objectification among women, and Internet addiction, as well as the psychological mechanisms underlying this relationship. The findings supported our proposed theoretical model. Higher levels of body surveillance predicted greater appearance anxiety, lower self-control, and more severe Internet addiction, with appearance anxiety and self-control serially mediating the link between body surveillance and Internet addiction. These findings have significant implications for women coping with excessive body surveillance and offer practical implications for clinical practice surrounding women’s Internet addiction.

## Data Availability

All data and materials are available upon reasonable request.
